# Genetic variants in bone morphogenetic proteins signaling pathway might be involved in palatal rugae phenotype in humans

**DOI:** 10.1038/s41598-021-92169-0

**Published:** 2021-06-16

**Authors:** Alice Corrêa Silva-Sousa, Guido Artemio Marañón-Vásquez, Maria Bernadete Sasso Stuani, Peter Proff, Kesly Mary Ribeiro Andrades, Flares Baratto-Filho, Mírian Aiko Nakane Matsumoto, Eva Paddenberg, Erika Calvano Küchler, Christian Kirschneck

**Affiliations:** 1 Department of Restorative Dentistry, School of Dentistry of Ribeirão Preto, USP-University of São Paulo, Ribeirão Preto, SP Brazil; 2 Department of Pediatric Dentistry and Orthodontics, School of Dentistry, Federal University of Rio de Janeiro, Rio de Janeiro, RJ Brazil; 3 Department of Pediatric Dentistry, School of Dentistry of Ribeirão Preto, USP-University of São Paulo, Ribeirão Preto, SP Brazil; 4 Department of Orthodontics, University of Regensburg, Franz-Josef-Strauss-Allee 11, 93053 Regensburg, Germany; 5 Department of Operative Dentistry, School of Dentistry, Univille–University from the Joinville Region, Joinville, Santa Catarina Brazil

**Keywords:** Genetics, Genetics research

## Abstract

This study investigated, if genetic variants in *BMP2, BMP4* and *SMAD6* are associated with variations in the palatal rugae pattern in humans. Dental casts and genomic DNA from 75 patients were evaluated. Each patient was classified as follows: total amount of rugae; bilateral symmetry in the amount, length and shape of the palatal rugae; presence of secondary or fragmentary palatal rugae; presence of unifications; predominant shape; and predominant direction of the palatal rugae. The genetic variants in *BMP2* (rs1005464 and rs235768), *BMP4* (rs17563) and *SMAD6* (rs2119261 and rs3934908) were genotyped. Genotype distribution was compared between palatal rugae patterns using the chi-square test (alpha = 0.05). The allele A was associated with the presence of secondary or fragmentary rugae for rs1005464 (OR = 2.5, 95%CI 1.1–6.3; p = 0.014). Secondary or fragmentary rugae were associated with the G allele in rs17563 (OR = 2.1, 95%CI 1.1–3.9; p = 0.017). rs17563 was also associated with rugae unification (p = 0.017 in the additive model). The predominant shape (wavy) was associated with rs2119261 (p = 0.023 in the additive model). The left–right symmetry of the length of primary rugae was associated with rs3934908 in the recessive model (OR = 3.6, 95%CI 1.2–11.7; p = 0.025). In conclusion, genetic variants in the BMP pathway impacted on palatal rugae pattern.

## Introduction

Palatal rugae are irregular structures located at the palatal mucosa in the oral cavity^1^. The term palatal rugae refers to a series of ridges produced by the folding of the wall of an organ. They are irregularly elevated ridges on the mucous membrane covering the anterior third part of the hard palate. Palatal rugae radiate transversely from the incisive papilla and the anterior part of the palatine raphe on either side^2^. Development of palatal rugae occurs during embryogenesis via the interaction of epithelium and mesenchyme tissues. Their morphology remains stable during life^3,4^, however varies from individual to individual^5–7^. Thus it is generally accepted that the individual genetic background impacts on this variability^8^.

Bone morphogenetic proteins 2 and 4 (*BMP2* and *BMP4* respectively) are genes that encode a secreted ligand of the transforming growth factor-beta superfamily (TGF-beta). This family of ligands bind TGF-beta receptors leading to the recruitment and activation of *SMAD* (mothers against decapentaplegic homolog) family transcription factors that regulate gene expression. *SMAD6* is a protein-coding gene member of the *SMAD* family, which mediates TGF-beta and *BMP* activity^9–11^.

*BMPs* are recognized as important participants in craniofacial development and in palatogenesis^12,13^. *BMP*s are known to present as a sign of mesenchymal proliferation related to palatogenesis^12,14^. *BMP4* induces the expression of Shh, which will induce the expression of *BMP2* that will positively regulate cell proliferation^13,15^. *BMP*s and *SMAD* can be found in the process of palatal rugae development^16,17^. Our previous study observed an association between genetic variants in *WNT11* and *WNT3* and variations in the pattern of palatal rugae in humans^8^. Interestingly, a reduction in palatal rugae in vivo was attributed to the overexpression of Shh^17^ that interacts with Wnt during palatogenesis^18,19^. Therefore, it is reasonable to hypothesize that variants in genes encoding *BMP*s and *SMAD*s also contribute to the determination of palatal rugae pattern. Therefore, this is the first study to investigate, if genetic variants in *BMP2, BMP4* and *SMAD6* are associated with variations in the palatal rugae pattern in humans.

## Results

Genotype distributions were within the Hardy–Weinberg equilibrium (data not shown). Thirty-eight (50.6%) patients were men and 37 (49.4%) were women. Their age ranged from 10 to 40 years and they were in mixed or permanent dentition. The phenotype distribution according to gender is presented in Table 1. There were no significant gender differences between groups (p > 0.05).

**Table 1:** Distribution of palatal rugae patterns according to gender (n = 75).

Pattern	Phenotype	Total n (%)	Male n (%)	Female n (%)	p-value
Total amount of rugae	< 8 rugae	34 (45.3)	18 (47.4)	16 (43.2)	0.817
	≥ 8 rugae	41 (54.7)	20 (52.6)	21 (56.8)	
Left–right symmetry regarding the amount of rugae	Symmetry	29 (38.7)	14 (36.8)	15 (40.5)	0.814
	Asymmetry	46 (61.3)	24 (63.2)	22 (59.5)	
Left–right symmetry regarding the length of primary rugae	Symmetry	22 (29.3)	9 (23.7)	13 (35.1)	0.318
	Asymmetry	53 (70.7)	29 (76.3)	24 (64.9)	
Secondary or fragmentary rugae	Present	43 (57.3)	21 (55.3)	22 (59.5)	0.816
	Absent	32 (42.7)	17 (44.7)	15 (40.5)	
Rugae unification	Present	49 (65.3)	25 (65.8)	24 (64.9)	> 0.999
	Absent	26 (34.7)	13 (34.2)	13 (35.1)	
Predominant shape	Curved	24 (32.0)	10 (26.3)	14 (37.8)	0.372
	Wavy	50 (66.7)	27 (71.1)	23 (62.2)	
	Straight	1 (1.3)	1 (2.6)	0 (0.0)	

Genotype distributions according to the palatal rugae patterns are presented in Table 2. For rs1005464 in *BMP2* the allele distribution was associated with the presence of secondary or fragmentary rugae. Individuals carrying the A allele had an increased chance to present secondary fragmentary rugae (OR = 2.5, 95%CI 1.1–6.3; p = 0.014). Similarly, in the dominant model (AA + AG *vs.* GG) individuals carrying the allele A had a three times higher chance to present secondary or fragmentary rugae (OR = 3.0, 95%CI 1.1–7.6; p = 0.037). The presence of secondary or fragmentary rugae was also associated with rs17563 in *BMP4*. In the allele distribution, carrying the G allele increased the chance to present secondary fragmentary palatal rugae (OR = 2.1, 95%CI 1.1–3.9; p = 0.017). In the recessive model (GG *vs.* AA + AG) individuals carrying the GG genotype had a more than five times higher chance to present secondary fragmentary rugae (OR = 5.5, 95%CI 1.3–26.1; p = 0.037). rs17563 was also associated with palatal rugae unification in the genotype distribution in the additive model (p = 0.017).

**Table 2:** Genotype and allele distributions for genetic variants in *BMP2* (rs1005464 e rs235768), *BMP4* (rs17563) and *SMAD6* (rs2119261 e 3934908) according to palatal rugae patterns.

Gene (and genetic variant)	Total amount of rugae		Left–right symmetry on the amount of the rugae		Left–right symmetry on the length of primary rugae		Secondary of fragmentary rugae		Rugae unification		Predominant shape		
	< 8 rugae (n = 34)	≥ 8 rugae (n = 41)	Symmetry (n = 29)	Asymmetry (n = 46)	Symmetry (n = 22)	Asymmetry (n = 52)	Absent (n = 32)	Present (n = 43)	Absent (n = 24)	Present (n = 51)	Curved (n = 24)	Wavy (n = 49)	Straight (n = 01)
**BMP2 (rs1005464)**													
AA	2 (6.3)	2 (5.0)	2 (7.4)	2 (4.4)	0 (0.0)	4 (8.0)	0 (0.0)	4 (9.8)	1 (4.2)	3 (6.3)	3 (12.5)	1 (2.1)	0 (0.0)
AG	7 (21.9)	15 (37.5)	7 (25.9)	15 (33.3)	8 (36.4)	14 (28.0)	7 (22.6)	15 (36.6)	9 (37.5)	13 (27.1)	7 (29.2)	15 (31.3)	0 (0.0)
GG	23 (71.8)	23 (57.5)	18 (66.7)	28 (62.2)	14 (63.6)	32 (64.0)	24 (77.4)	22 (53.6)	14 (58.3)	32 (66.6)	14 (58.3)	32 (66.6)	0 (0.0)
p-value genotype	0.359		0.732		0.348		0.057		0.648		0.190		
p-value allele	0.335		0.915		0.603		0.014*		0.663		0.191		
**BMP2 (rs235768)**													
AA	1 (2.9)	1 (2.5)	1 (3.5)	1 (2.2)	0 (0.0)	2 (3.8)	1 (3.2)	1 (2.3)	0 (0.0)	2 (4.1)	0 (0.0)	2 (4.1)	0 (0.0)
AT	16 (47.1)	23 (57.5)	15 (51.7)	24 (53.4)	11 (50.0)	28 (53.9)	16 (51.6)	23 (53.5)	13 (50.0)	26 (54.2)	12 (50.0)	27 (55.1)	0 (0.0)
TT	17 (50.0)	16 (40.0)	13 (44.8)	20 (44.4)	11 (50.0)	22 (42.3)	14 (45.2)	19 (44.2)	13 (50.0)	20 (41.7)	12 (50.0)	20 (40.8)	1 (100.0)
p-value genotype	0.668		0.947		0.578		0.965		0.496		0.620		
p-value allele	0.523		0.956		0.479		0.996		0.424		0.468		
**BMP4 (rs17563)**													
GG	4 (12.5)	9 (23.1)	4 (14.8)	9 (20.5)	4 (18.2)	9 (18.4)	2 (6.5)	11 (27.5)	6 (25.0)	7 (14.9)	4 (16.7)	9 (19.2)	0 (0.0)
AG	13 (40.6)	18 (46.2)	13 (48.2)	18 (40.9)	8 (36.4)	23 (46.9)	14 (45.2)	17 (42.5)	8 (33.3)	23 (48.9)	8 (33.3)	23 (48.9)	0 (0.0)
AA	15 (46.9)	12 (30.8)	10 (37.0)	17 (38.6)	10 (45.5)	17 (34.7)	15 (48.4)	12 (30.0)	10 (41.7)	17 (36.2)	12 (50.0)	15 (31.9)	0 (0.0)
p-value genotype	0.301		0.777		0.653		0.054		0.017*		0.315		
p-value allele	0.106		0.811		0.538		0.017*		0.791		0.237		
**SMAD6 (rs2119261)**													
TT	4 (12.5)	4 (9.8)	2 (7.4)	6 (13.0)	4 (18.2)	4 (7.8)	4 (12.5)	4 (9.8)	1 (4.2)	3 (5.9)	6 (25.0)	2 (4.1)	0 (0.0)
CT	22 (68.8)	21 (51.2)	17 (63.0)	26 (56.6)	14 (63.6)	29 (56.9)	21 (65.6)	22 (53.7)	14 (58.3)	35 (68.6)	11 (45.8)	32 (65.3)	0 (0.0)
CC	6 (18.8)	16 (39.0)	8 (29.6)	14 (30.4)	4 (18.2)	18 (35.3)	7 (21.9)	15 (36.6)	9 (37.5)	13 (25.5)	7 (29.2)	15 (30.6)	0 (0.0)
p-value genotype	0.172		0.734		0.211		0.396		0.560		0.023*		
p-value allele	0.159		0.774		0.121		0.286		0.419		0.171		
**SMAD6 (rs3934908)**													
TT	7 (20.6)	8 (20.0)	6 (20.7)	9 (20.0)	8 (36.4)	7 (13.5)	9 (29.0)	6 (14.0)	6 (23.1)	9 (18.8)	9 (37.5)	6 (12.2)	0 (0.0)
CT	16 (47.1)	22 (55.0)	13 (44.8)	25 (55.6)	8 (36.4)	30 (57.7)	15 (48.4)	23 (53.5)	11 (42.3)	27 (56.2)	10 (41.7)	27 (55.1)	1 (100.0)
CC	11 (32.4)	10 (25.0)	10 (34.5)	11 (24.4)	6 (27.3)	15 (28.8)	7 (22.6)	14 (32.6)	9 (34.6)	12 (25.0)	5 (20.8)	16 (32.7)	0 (0.0)
p-value genotype	0.748		0.598		0.067		0.253		0.511		0.116		
p-value allele	0.680		0.577		0.172		0.131		0.758		0.106		

* and bold indicate statistical significance difference (p < 0.05).

The predominant shape was associated with rs2119261 in *SMAD6* in the genotype distribution (p = 0.023). The left–right symmetry on the length of primary rugae was associated with rs3934908 in *SMAD6* only in the recessive model (TT *vs.* CT + TT) with individuals carrying the TT genotype having a higher chance to present symmetry of palatal rugae (OR = 3.6, 95%CI 1.2–11.7; p = 0.025).

## Discussion

The development of the mammalian palate, including palatal rugae formation, is a complex process. It is characterized as a multi-step process that includes mesenchymal cell proliferation, palatal shelf outgrowth, elevation, fusion and eventually disappearance of the midline epithelial seam^12^. Palatal rugae are conserved in all mammals, including humans and rodents. Although the number and patterns of palatal rugae are species-specific, previous studies in animal models clearly support that palatal rugae development is under strict genetic control and many different genes interact in order to establish a specific palatal rugae pattern^16,17^. Therefore, in the present study we explored for the first time, if genetic variants in *BMP* signaling pathways are involved in palatal rugae variation in humans.

Fragmentary and secondary rugae are those with smaller length. The presence of these rugae is frequently observed in patients, particularly in the posterior half of the rugae area. In our study, the intronic variant rs1005464 in *BMP2* and the missense variant rs17563 in *BMP4* were associated with the presence of fragmentary or secondary rugae. A previous study in rodents showed that palatal rugae are sequentially added to the growing palate, which seems to be dependent on activation and inhibition mechanisms in the palate. This study from 2008 proposed that *BMP*s should be further investigated^20^. Later, a study by Kawasaki et al.^17^ observed that the reduction in palatal rugae was related to the overexpression of Shh signaling. Shh regulates the expression of the *BMP2* and *BMP4* in the palatal mesenchyme^21^.

Both genetic variants associated with fragmentary and secondary palatal rugae were previously associated with other oral phenotypes. The genetic variant rs1005464 in *BMP2* has been associated with a variety of conditions including dental crowding^22^ and mandibular retrognathism^23^. *BMP4* is a widely studied gene as an etiologic factor involved in oral cleft establishment, which is a congenital alteration in palate formation. A literature review demonstrated that many studies in different populations observed the association between rs17563 in *BMP4* and non-syndromic oral clefts^24^. These highlight the important role of this genetic variant in palate formation and the determination of palatal patterns. In the genetic variant rs17563 the polymorphic allele replaces the amino acid valine with alanine at position 152 of the protein, however, the structure and function of the protein are not significantly affected by the substitution^25^. In our study, subjects carrying the alanine form had an increased chance to present fragmentary/secondary rugae and rugae unification.

*SMAD*6 belongs to the *SMAD* family of proteins, which constitutes an important signaling pathway regulating the transcription of genes of the Transforming Growth Factor β (TGF-β) family, including *BMPs*^26^. *SMAD6* inhibits *BMP* signaling in the nucleus via the interaction with transcription repressors^27^. Therefore, we hypothesize that genetic variants in *SMAD6* are candidates for variations in the palatal rugae pattern. Indeed, the genetic variant rs2119261 was associated with the predominant palatal rugae shape (curved or wavy), while rs3934908, also in *SMAD6*, was associated with left–right symmetry of rugae.

In our first study, we observed that genetic variants in *WNT3A* and *WNT11* were associated with the left–right asymmetry regarding the amount of palatal rugae^8^. In animal models the ablation of WNT signaling in the oral epithelium blocked the formation of palatal rugae^28^. Interaction between WNT signaling and *BMP*/*SMAD* signaling has been widely explored, including in the context of craniofacial and dental formation^29,30^. In the present study, genetic variants in *BMP*/*SMAD*-signaling-encoding genes were associated with palatal rugae. Thus, this is the first study to suggest that the variations in *BMP*/*SMAD* are involved in the development of palatal rugae in humans and also impact on anatomic variations among individuals. Interestingly, this pathway is well known involved in tooth formation and palatal rugae seem to have a different pattern in individuals with congenitally missing teeth^31^.

The development of the secondary palate has been an important topic in craniofacial research, as its failure results in oral clefting (a common birth defect). However, the mechanisms involved in palatal rugae pattern in subjects without birth defects received little attention so far. Our sample size is an obvious limitation of our study. Although it brings important novel preliminary information, future multicentric studies with larger samples are necessary to replicate our results and to unravel the genetic background of the palatal rugae pattern in humans. Briefly, variants in genes encoding for the BMP/SMAD signaling pathway might be involved in the determination of the palatal rugae pattern in humans. Also, more genetic variants covering BMPs should be evaluated in future studies.

## Methods

The protocol of this cross-sectional study was approved by the Research Ethics Committee of the School of Dentistry of Ribeirão Preto, University of São Paulo (3.150.551). This study was conducted after the approval of the Institutional Ethics Committee and all experiments were performed in accordance with latest version of Declaration of Helsinki guidelines. All the patients gave written informed consent after the nature of the research procedures had been fully explained to them. If the patient were under 18, an informed consent from a parent and/or legal guardian and an assent from the patient was also obtained.

This study was conducted following the Strengthening the Reporting of Genetic Association study (STREGA) statement checklist^32^. The sample size was determined based on our previous study^8^. Briefly, 75 biologically unrelated patients (one subject per family), who were undergoing orthodontic treatment at the School of Dentistry of Ribeirão Preto from 2017 to 2018, were included. Patients with craniofacial congenital anomalies or syndromes, oral clefts, severe transverse maxillary deficiency, palatal asymmetry, scar tissue, previous orthodontic treatment or with poor quality records were excluded from the study. Availability of pretreatment dental casts free of voids and air bubbles and good quality intraoral occlusal photographs was also a prerequisite.

### Determination of the palatal rugae pattern

Palatal rugae were evaluated by a single evaluator via direct visual screening of the casts and intraoral occlusal photography of each patient. Information on rugae characteristics was initially recorded in a rugoscopy chart^33^ and rugae classified according to:

1. Length: Primary (≥ 5 mm), secondary (3–5 mm), and fragmentary (2–3 mm)^34^,
2. Shape: Curved, wavy, straight and circular^35^,
3. Direction: Forwardly directed, backwardly directed and perpendicular^36–38^,
4. Unification: Divergent and convergent^36–38^.

Rugae length measurements were performed directly from dental casts using an electronic hand-held digital caliper (Digimatic CD-15DCX; Mitutoyo^®^, Kawasaki, Japan). For non-straight rugae, a segment of wire was adapted according to the shape of the rugae, and then it was rectified for measurement. More details regarding pattern definition and measurements are described in Silva-Sousa et al.^8^.

Based on the rugae characterization, individuals were classified according to their rugae patterns, as presented in Table 3.

**Table 3:** Classification parameters for differentiating palatal rugae patterns.

(1) Total amount of rugae	Less than 8 rugae Equal or more than 8 rugae
(2) Left–right symmetry regarding the amount of the rugae	Symmetry—equal amount of rugae to the left and right sides of the median palatal raphe Asymmetry—different amount of rugae to the left and right sides of the median palatal raphe
(3) Left–right symmetry regarding the length of primary rugae	Symmetry—no difference or difference up to 0.5 mm between the mean right and left primary rugae lengths Asymmetry—greater than 0.5 mm difference between the mean right and left primary rugae lengths
(4) Left–right symmetry of rugae shape	Symmetry—presence of the same rugae shapes on both sides Asymmetry—different rugae shapes on both sides
(5) Secondary or fragmentary rugae	Presence Absense
(6) Rugae unifications	Presence Absense
(7) Predominant shape	Curved Wavy Straight
(8) Direction of the rugae according to Carrea’s classification^36^	Only forwardly directed rugae Only perpendicular rugae Only backwardly directed rugae Differently directed rugae

Five randomly chosen dental casts were evaluated twice within an 8-week interval, and then Cohen’s kappa (κ) was applied to check the intraexaminer coefficient of agreement. The intraclass correlation coefficient (ICC) was used to determine the rater consistency of the repeated evaluations of rugae length. All ICC and κ values were above 0.9 (p < 0.001).

### Genotyping

Genomic DNA extracted from oral cells isolated from saliva samples of all included patients was used for the allelic discrimination analysis, as previously described in Küchler et al.^39^. Evaluation of DNA amount and purity was performed by spectrophotometery (Nanodrop 1000; Thermo Scientific, Wilmington, DE). Genetic variants in *BMP2* (rs1005464 and rs235768), *BMP4* (rs17563) and *SMAD6* (rs2119261 and rs3934908) were blindly genotyped by real-time polymerase chain reactions (PCR) using TaqMan assay (Step One Plus Real-Time PCR System, Applied Biosystems, Foster City, CA). The characteristics of the assessed genetic variants are presented in Table 4.

**Table 4:** Characteristics of the selected genetic variants.

Gene	Initials	Genetic variant	Base change	Function	Global MAF	PCR amplification rate (%)
Bone morphogenetic protein 2	*BMP2*	rs1005464	A/G	Intron	0.78	96.0
		rs235768	A/T	Missense (Arg > Ser)	0.77	98.6
Bone morphogenetic protein 4	*BMP4*	rs17563	A/G	Missense (Val > Ala)	0.67	94.6
SMAD family member 6	*SMAD6*	rs2119261	C/T	Intron	0.54	97.3
		rs3934908	C/T	Intron	0.59	98.6

MAF means minor allele frequency. Data obtained from databases: https://www.ncbi.nlh.nih.gov/snp/; http://genome.uscs.edu/; and, https://www.thermofisher.com/.

Arg means Arginine, Ser means Serine, Val means Valine, and Ala means alanine.

### Statistical analysis

Chi-square tests were used to compare the genotype/allele distribution in additive, dominant and recessive models between the different palatal rugae patterns. Odds ratios (OR) and their 95% confidence intervals (CI) were calculated to evaluate the chance of presenting certain rugae phenotypes for the genetic variants. In the analysis of each genetic variant, cases with missing values were dropped from the analysis. The Hardy–Weinberg equilibrium was also evaluated by Chi-square test. All analyses were performed using two-tailed tests (α = 0.05) on Epi Info 7.0.
